# Vegetation Structure Index (VSI): Retrieving Vegetation Structural Information from Multi-Angular Satellite Remote Sensing

**DOI:** 10.3390/jimaging7050084

**Published:** 2021-05-09

**Authors:** Ram C. Sharma

**Affiliations:** Department of Informatics, Tokyo University of Information Sciences, 4-1 Onaridai, Wakaba-ku, Chiba 265-8501, Japan; sharma@rsch.tuis.ac.jp; Tel.: +81-43-236-4603

**Keywords:** forests, structure, biomass, BRDF, MODIS, multi-angular, NDVI (fore-back), vegetation structure index

## Abstract

Utilization of the Bidirectional Reflectance Distribution Function (BRDF) model parameters obtained from the multi-angular remote sensing is one of the approaches for the retrieval of vegetation structural information. In this research, the potential of multi-angular vegetation indices, formulated by the combination of multi-spectral reflectance from different view angles, for the retrieval of forest above-ground biomass was assessed in the New England region. The multi-angular vegetation indices were generated by the simulation of the Moderate Resolution Imaging Spectroradiometer (MODIS) BRDF/Albedo Model Parameters Product (MCD43A1 Version 6)-based BRDF parameters. The effects of the seasonal (spring, summer, autumn, and winter) composites of the multi-angular vegetation indices on the above-ground biomass, the angular relationship of the spectral reflectance with above-ground biomass, and the interrelationships between the multi-angular vegetation indices were analyzed. Among the existing multi-angular vegetation indices, only the Nadir BRDF-adjusted NDVI and Hot-spot incorporated NDVI showed significant relationship (more than 50%) with the above-ground biomass. The Vegetation Structure Index (VSI), newly proposed in the research, performed in the most efficient way and explained 64% variation of the above-ground biomass, suggesting that the right choice of the spectral channel and observation geometry should be considered for improving the estimates of the above-ground biomass. In addition, the right choice of seasonal data (summer) was found to be important for estimating the forest biomass, while other seasonal data were either insensitive or pointless. The promising results shown by the VSI suggest that it could be an appropriate candidate for monitoring vegetation structure from the multi-angular satellite remote sensing.

## 1. Introduction

Forests have experienced dramatic changes in terms of cover, density, and biomass worldwide. Monitoring of forest biomass and carbon stock changes is vital to comprehend deforestation and degradation conditions that have implications for the climate system. At local scales, forest structural parameters such as diameter at breast height, canopy height, etc., can be obtained from the measurement of individual trees. Then, the forest biomass can be estimated with allometric functions, which provides a functional relationship with easily measured variables such as standing tree height and diameter at breast height [1,2]. However, satellite remote sensing is an expected technology for upscaling the in situ estimates of forest biomass into broad scales.

Multi-angular remote sensing refers to the observation of surface reflectance from multiple view angles beyond nadir alone with the account of solar position as well. It provides a technique of measuring anisotropic surface reflectance of the land surface. The anisotropic reflectance, the directional dependency of the reflectance with sun-sensor geometry, is a unique characteristic of the land surface [3,4,5,6]. Researchers have developed some Bidirectional Reflectance Distribution Function (BRDF) models such as computer simulation [7], empirical [8], physical radiative transfer [9], physical geometric optical [10,11], and semi-emperical [12,13] to describe the anisotropic characteristics of the land surface. Since the bidirectional reflectance is sensitive to vegetation structure (canopy cover, height, volume, etc.) [14,15], it can be one of the approaches for the retrieval of above-ground biomass. The multi-angular remote sensing has also been utilized for characterization of biomes [16], forests [17], agricultural landscape [18], vegetation physiognomic types [19], and chemical attributes of the canopy [20,21]. On the other hand, the angular variation adds noise and uncertainty on the study of land surface biophysical parameters such as land cover and vegetation classification and phenology [22,23,24]. The BRDF models have many applications such as normalizing the BRDF effects of images taken at multiple sun-sensor geometry, estimation of albedo by integration of multi-angular reflectance, and retrieval of land surface attributes by the interpretation of BRDF shapes [25,26,27]. Therefore, more observations and research in the field of BRDF are necessary for better understanding the land-surface anisotropic characteristics and accurate retrieval of structural information. 

For the retrieval of vegetation biophysical parameters from the multi-angular remote sensing, different approaches such as radiative transfer modeling [7], geometric-optical modeling [10], spectral invariant [28,29] and BRDF model parameters [30,31] have been attempted by the researchers. The Ross-Thick/Li-Sparse-Reciprocal (RTLSR) is one of the semi-empirical BRDF models. In this model, the bi-directional reflectance (R) is described for a given sun zenith angle (SZA), view zenith angle (VZA), and relative azimuth angle (RAA) with isotropic, volumetric and geometric scattering coefficients and the kernels for volumetric scattering and geometric scattering [32]. Radiative transfer models are effective to estimate the biophysical and biochemical properties of the forest canopy. However, utilization of multi-angular indices as the combination of spectral reflectance measured from multiple view angles can be an uncomplicated and straightforward technique to derive vegetation structure information. An overview of the multi-angular indices available in the literature has been presented in Table 1.

Derivation of more sensitive vegetation indices from multi-angular remote sensing data is important for better retrieval of the vegetation structural information. The major objectives of this research are to assess the potential of multi-angular vegetation indices for the retrieval of forest above-ground biomass, and to propose a more sensitive vegetation index for the estimation of above-ground biomass. The effects of seasonal composites of the multi-angular vegetation indices on above-ground biomass, angular relationship of the spectral reflectance with above-ground biomass, and the interrelationships between the multi-angular vegetation indices have also been discussed.

## 2. Materials and Methods

### 2.1. Study Areas and In Situ Data

**Figure 1:**
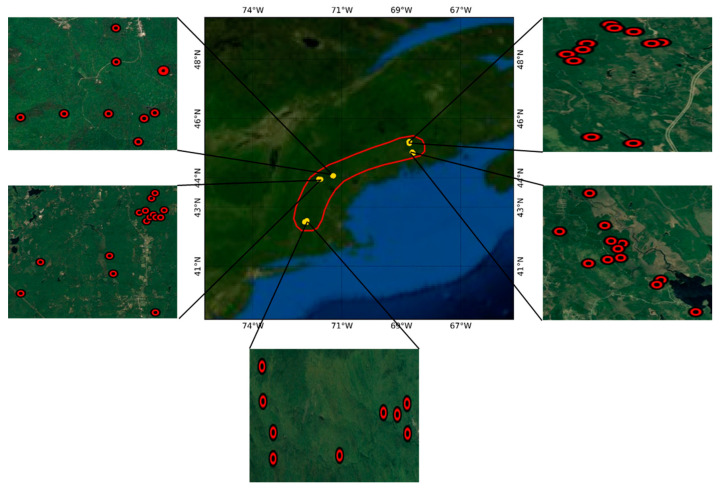
Location of the study sites and distribution of the sample plots.

This research was implemented in the New England region where a high-quality forest inventory database is available [38]. The database constitutes the field measurements conducted in five forests in 2009. It includes forest inventory data from five forests: Harvard Forest (moist temperate), Howland Research Forest (mature evergreen), Hubbard Brook Experimental Forest (deciduous hardwoods), Bartlett Experimental Forest (deciduous hardwoods), and Penobscot Experimental Forest (mixed conifers and hardwoods). The above-ground biomass was calculated with diameter at breast height greater than 10 cm using the allometric function [1]. The heterogeneous sample plots mixed with other land cover types, such as built-up areas, water ponds, etc., were discarded from the analysis. Among 59 sample plots (1 ha size) available, 55 sample plots were selected for this analysis. Location map of the study sites and distribution of sample plots have been shown in Figure 1.

### 2.2. Proposal of New Multi-Angular Indices

A typical forest canopy is composed of green canopy cover (sunlit crown and shadowed crown) and canopy shadow fraction (shadowed ground and shadowed crown). The fractional area of the canopy components vary significantly in the principal plane with respect to the view zenith angles [39]. The geometric configuration of (sun zenith angle, view zenith angle, and relative azimuth angle) has been used to define the concerned sun-sensor geometries, such as Nadir-sun and Nadir-view (0°, 0°, 0°), Hot-spot (45°, 45°, 0°), Nadir (45°, 0°, 0°), and Dark-spot (45°, 45°, 180°). While the scene viewed from the back-scattering direction is mostly composed of sunlit ground and sunlit crown, the shadowed crown and shadowed ground dominate the scene viewed from the fore-scattering direction. Red reflectance at the Back-scattering direction is sensitive to hiding of the ground which should be faster in tall and dense (high-biomass) canopy [40]. The larger the ground surface hidden by canopies, the lower the red reflectance at the Back-scattering direction. For tall and dense canopy, near infrared reflectance at the Fore-scattering direction should be higher due to the effect of leaf area index changes. In this research assumed hot-spot (45°, 45°, 0°) and dark-spot (45°, 45°, 180°) were used as the Back-scattering and Fore-scattering directions, respectively. Fore-scattering (Fore) and Back-scattering (Back) Normalized Difference Vegetation Index ($NDVI_{Fore-back}$) can be calculated as the normalized difference between the Near Infrared (Nir) reflectance observed at Fore-scattering (Fore) direction and the Red reflectance (Red) observed at Back-scattering (Back) direction (Equation (1)) to be sensitive to the volumetric structure of the forest canopy. Then, by integrating the non-linear interaction of the vegetation coverage ratio, indicated by the term ($1-Nir_{Fore}$), the Vegetation Structure Index (VSI) has been proposed in the research (Equation (1)).

(1) $$VSI=\frac{NDVI_{Fore-back}}{1-Nir_{Fore}}$$

### 2.3. Processing of Satellite Data

The Moderate Resolution Imaging Spectroradiometer (MODIS) BRDF/Albedo Model Parameters Product (MCD43A1) has been providing BRDF model parameters based on the RTLSR model [33]. The BRDF model parameters (isotropic, volumetric and geometric coefficients) were obtained from the MODIS BRDF/Albedo product (MCD43A1 Version 6) of 2009. This dataset is produced daily using 16 days of Terra and Aqua MODIS data at a 500-m (m) resolution. Using the central geolocation point of each plot, the BRDF model parameters were extracted for a single MODIS pixel. Then, the bidirectional reflectance for the assumed Hot-spot (45°, 45°, 0°), Nadir (45°, 0°, 0°), and Dark-spot (45°, 45°, 180°) were calculated by using the BRDF model parameters (isotropic, volumetric and geometric scattering coefficients) and look-up values for the kernels of volumetric scattering and geometric scattering. The Back-scattering and Fore-scattering reflectance were obtained from the assumed Hot-spot (45°, 45°, 0°) and Dark-spot (45°, 45°, 180°) geometries, respectively. Seasonal median composites, spring (March–May), summer (June–August), autumn (September–November), and winter (December–February), were generated from the daily calculations of the multi-angular vegetation indices.

## 3. Results

### 3.1. Performance of Existing Multi-Angular Indices

**Figure 2:**
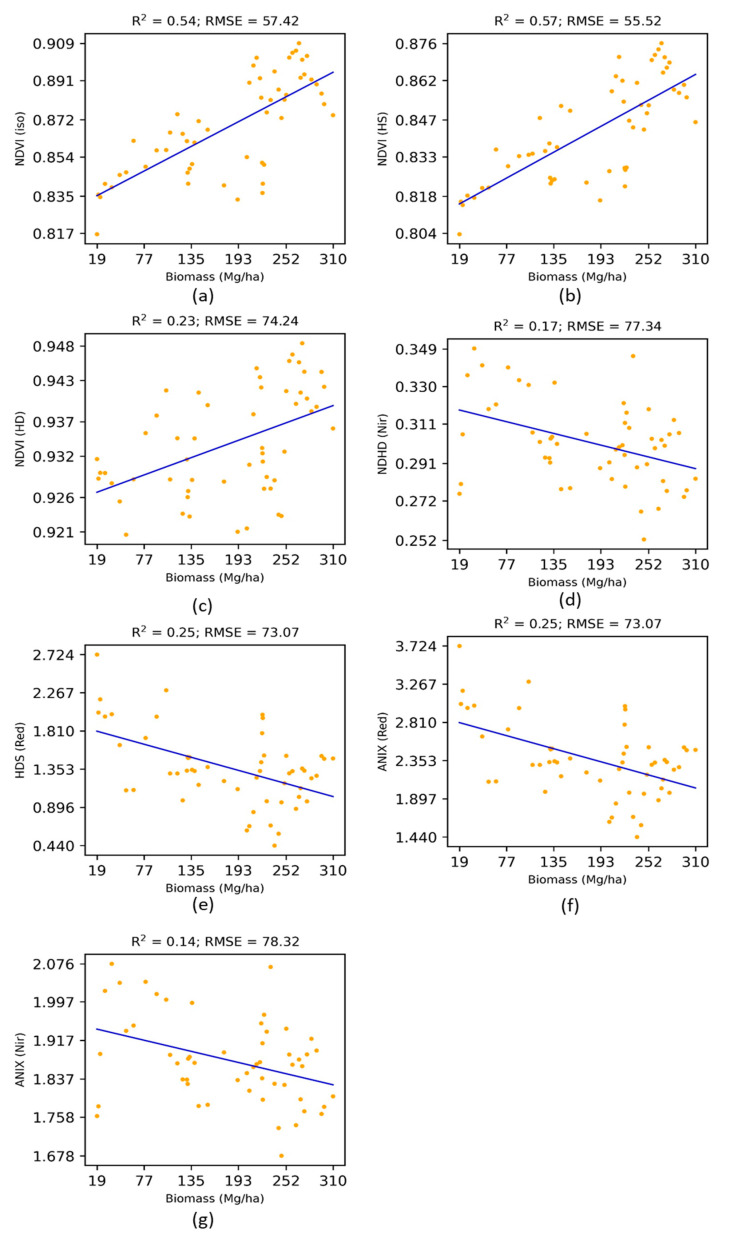
Performance of existing multi-angular vegetation indices for the estimation of above-ground biomass.

The performance of the multi-angular vegetation indices was assessed using linear regression analysis with the in situ above-ground biomass data in terms of Coefficient of determination (R^2^) and Root Mean Square Error (RMSE). The relationships between existing multi-angular spectral indices and above-ground biomass have been shown in Figure 2, and the results have also been summarized in Table 2.

As shown in Table 3, only the Nadir BRDF-adjusted NDVI ($NDVI_{iso})$ and Hot-spot-incorporated NDVI ($NDVI_{HS})$ showed a significant relationship (more than 50%) with the above-ground biomass. Other multi-angular vegetation indices, Anisotropy index ($ANIX_{Red}$), Anisotropy index ($ANIX_{Nir}$), Hot-spot dark spot index ($HDS_{red})$, Normalized difference between hot-spot and dark-spot index ($NDHD_{nir}$), and Hot-spot dark-spot NDVI ($NDVI_{HD}$) did not show sensitivity towards the above-ground biomass.

**Figure 3:**
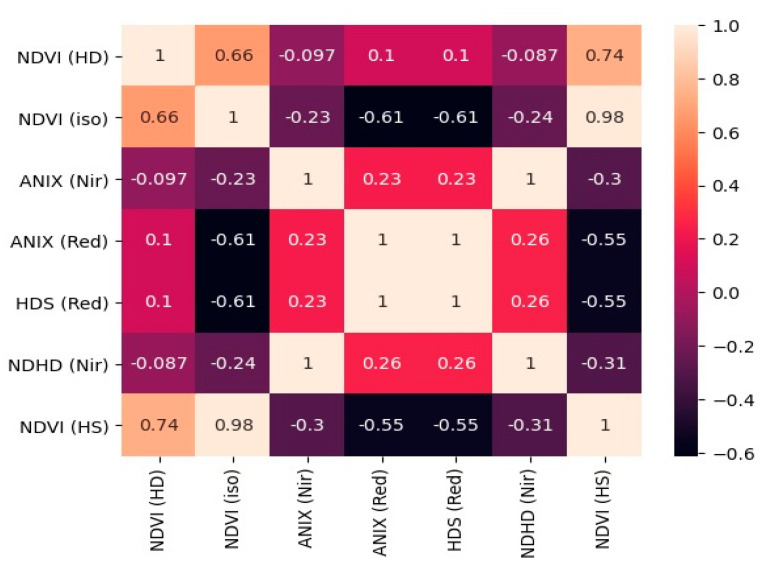
Correlation matrix of the extant multi-angular vegetation indices.

The correlation matrix of the extant multi-angular indices has been shown in Figure 3. Among the seven extant multi-angular indices, the highly correlated pairs were (i) $ANIX_{Nir}$ and $NDHD_{nir}\;$(ii)$\;ANIX_{Red}$ and $HDS_{red}\;$(iii) $NDVI_{iso}$ and ${NDVI}_{HS}$. This analysis confirms that only a few extant multi-angular indices are important for the estimation of above-ground biomass.

### 3.2. Performance of New Multi-Angular Indices

**Figure 4:**
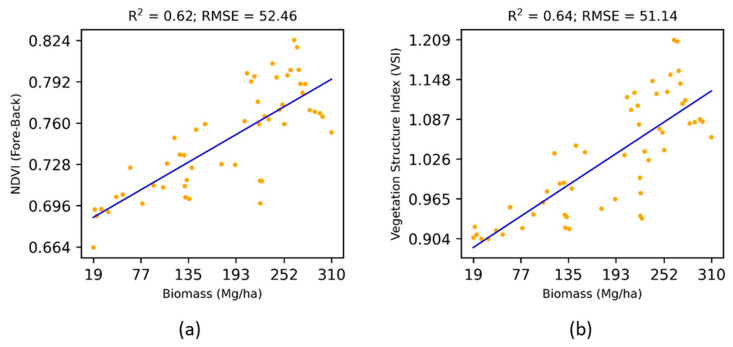
Performance of new multi-angular vegetation indices for the estimation of above-ground biomass.

The $NDVI_{Fore-back}$ showed higher sensitivity (R^2^ = 0.62, RMSE = 52.46) towards the above-ground biomass than existing multi-angular vegetation indices (Table 2). Furthermore, the Vegetation Structure Index (VSI) proposed in the research performed in the most efficient way explaining 64% variation (R^2^ = 0.64, RMSE = 51.14) of the above-ground biomass (Figure 4).

### 3.3. Effects of View Angles on Biomass

**Figure 5:**
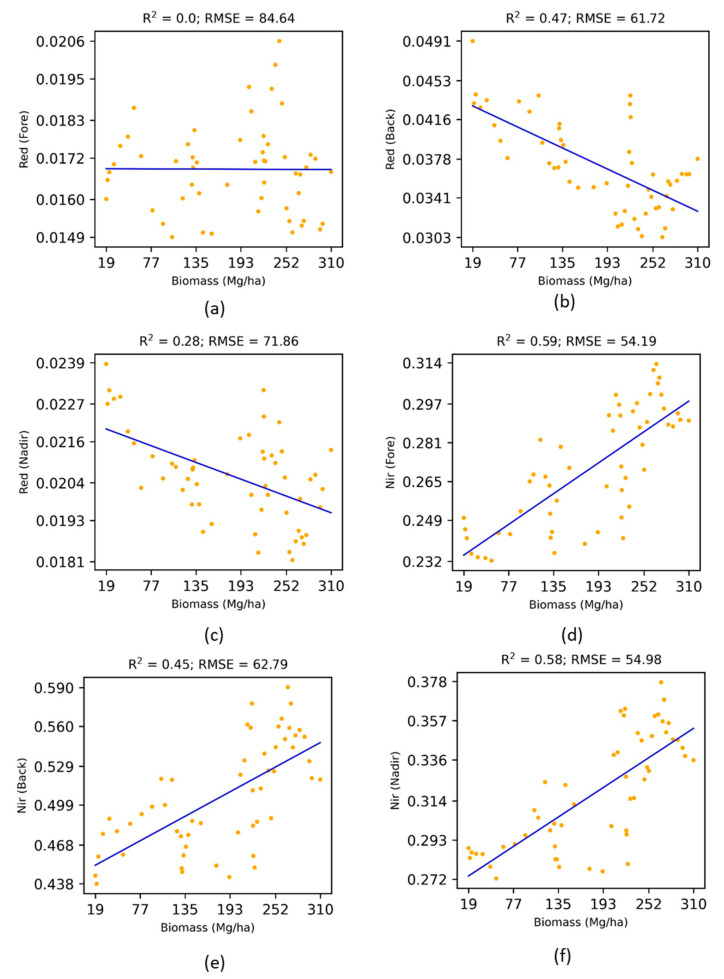
Effects of view angles on the above-ground biomass.

The effects of the view angles (Fore-scattering versus Back-scattering) on the above-ground biomass have been shown in Figure 5.

For the Red reflectance: the Back-scattering direction was found to be more sensitive (R^2^ = 0.47, RMSE = 61.72) to the above-ground biomass than the nadir direction, whereas the Fore-scattering direction was quite insensitive. Both the Back-scattering and Nadir reflectance were inversely proportional to the above-ground biomass. In contrast, for the Near Infrared reflectance, the Fore-scattering direction was found to be more sensitive (R^2^ = 0.59, RMSE = 54.19) to the above-ground biomass than the Back-scattering and Nadir directions. All three directions (Fore-scattering, Back-scattering, and Nadir) were directly proportional to the above-ground biomass. Therefore, the right choice of the spectral channel and observation geometry should be considered for improving the estimates of above-ground biomass. It should be noted that the $NDVI_{Fore-back}\;$ has been built by integrating the most sensitive spectral channel and observation geometry.

**Figure 6:**
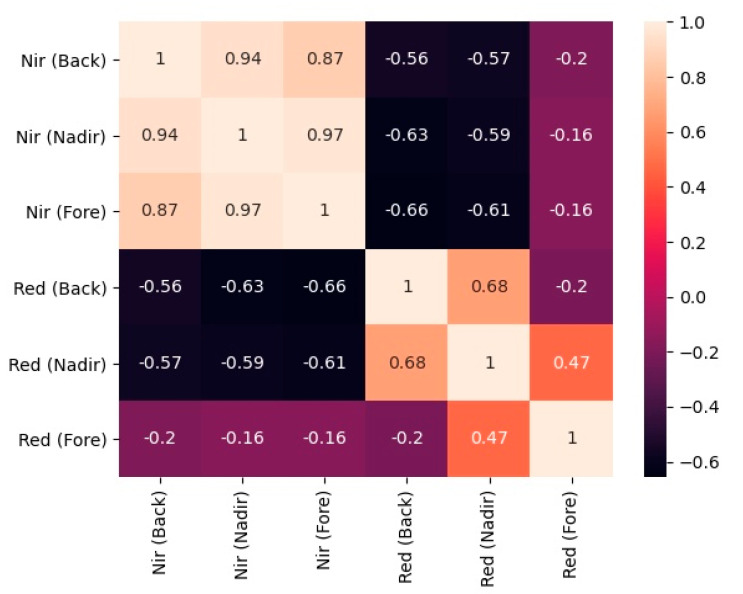
Correlation matrix of the reflectances measured at different angular configurations in the principle plane.

The correlation matrix of the red and near infrared reflectance measured at different angular configurations (Back-scattering, Nadir, and Fore-scattering) has been shown in Figure 6. The correlation matrix showed that near infrared reflectance was highly correlated across different angular configurations (Back-scattering, Nadir, and Fore-scattering) in the principle plane than the red reflectance.

### 3.4. Interrelationships between Structural Indices

**Figure 7:**
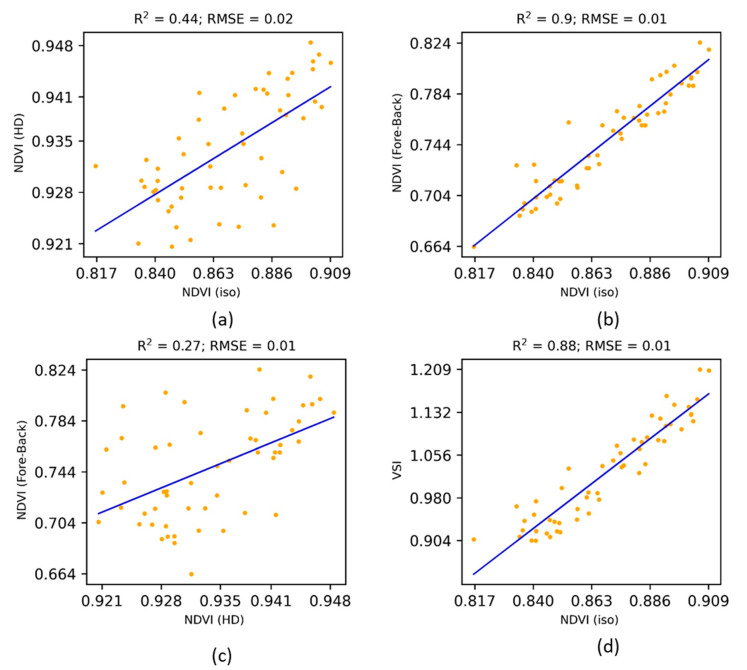
Interrelationships between multi-angular indices.

Figure 7 shows the interrelationships between multi-angular structural indices. $NDVI_{Fore-back}\;$ was more related to the $NDVI_{iso}$ than the $NDVI_{HD}.$ Nevertheless, $NDVI_{Fore-back}$ and $NDVI_{HD}$ were quite distinct with lower coefficient of determination (R^2^ = 0.27). The relationship between VSI and $NDVI_{iso}$ was lower (R^2^ = 0.88) than that of $NDVI_{Fore-back}$ and $NDVI_{iso}$. Still, the newly proposed index in the research was quite distinct from the existing indices while being more sensitive towards the above-ground biomass.

### 3.5. Effects of Seasonal Data on Biomass

**Figure 8:**
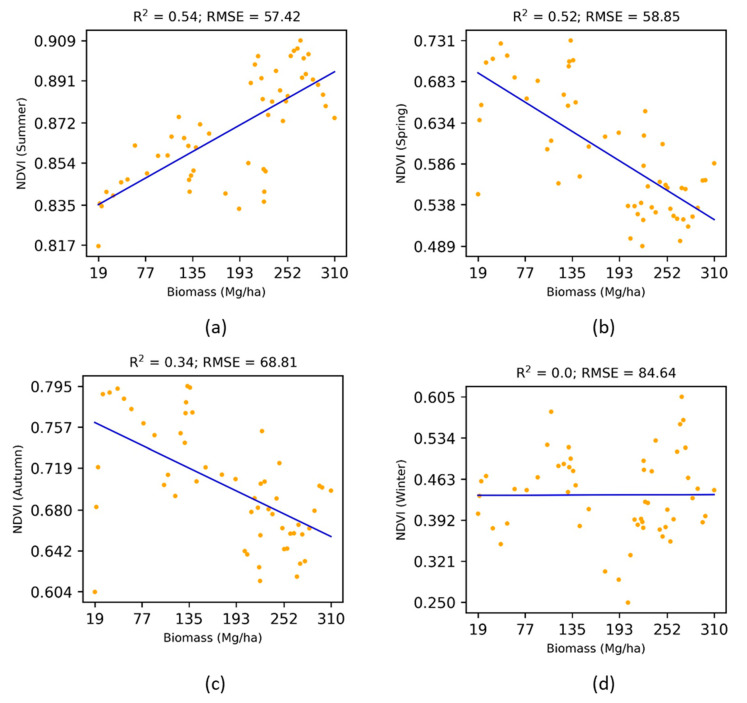
Effects of seasonal NDVI (iso) on the above-ground biomass.

**Figure 9:**
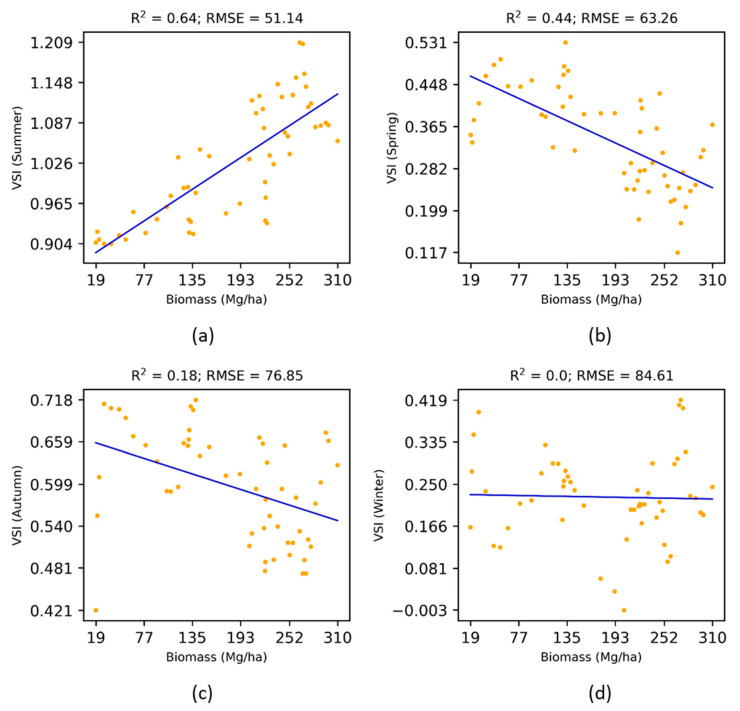
Effects of seasonal VSI on the above-ground biomass.

The analysis on above (Section 3.1, Section 3.2, Section 3.3 and Section 3.4) were based on median composites of the reflectance in the summer season. Figure 8 and Figure 9 show the seasonal effects of multi-angular vegetation indices $(NDVI_{iso}$ and VSI) on the above-ground biomass. Both the $NDVI_{iso}$ and VSI in summer were most sensitive to the above-ground biomass, whereas other seasons were either insensitive (winter season) or pointless (spring and autumn seasons with a decreasing trend). Therefore, the right choice of seasonal data was found to be important for estimating the forest biomass.

### 3.6. Statistical Significance Results

The D’Agostino and Pearson’s normality test and Shapiro–Wilk test were performed to confirm if the distribution of the available data is normal or not. The *p*-values for the D’Agostino and Pearson’s normality test and Shapiro–Wilk test were 0.019513 and 0.002633, respectively. Since the *p*-values of both tests were less than 0.05 (95% confidence), data were not distributed normally. Therefore, nonparametric statistical tests (Spearman’s rank correlation, Kendall’s rank correlation, and Kruskal–Wallis H-test) that do not assume a specific distribution to the data were performed to confirm significance of the linear regression coefficients reported. Associated *p*-values of the Spearman’s rank correlation and Kendall’s rank correlation have been shown in Table 3 for all independent variables concerned. Regardless of the low coefficient of determination (R^2^) values of the extant multi-angular indices (Table 2) and multi-angular reflectances (Figure 5) and the associated regression model, which could not explain much of the variation of data, all of them were significantly correlated to the above-ground biomass with *p*-value > 0.05 (95% confidence) except the Red reflectance at Fore-scattering direction (*p*-values = 0.564852, 0.692547). Nevertheless, the newly proposed Vegetation Structure Index (VSI) with highest R^2^ value and low *p*-value (0.000000) was able to explain much of the variation of the above-ground biomass data.

## 4. Discussion

There is an increased need for improved retrieval of canopy structural information from remote sensing data. The optical-imagery-based metrics have been useful to generate canopy structural attributes in different forests [41,42]. Radiative transfer models have shown efficient performance on the estimation of canopy gap fraction and biochemistry [43,44]. The multi-angular remote sensing datasets have a particular capability of capturing structural information and enable wider applications in ecology and terrestrial monitoring [45,46,47]. The spectral indices derived from multi-angular observations are promising techniques that can be used to obtain vegetation structural attributes [48,49,50].

Some previous studies have demonstrated the utility of multi-angular reflectance measurements for assessing vegetation structure such as leaf area index, canopy clumping, etc. [51,52]. In this research, we evaluated the performance of seven extant multi-angular vegetation indices for the estimation of above-ground biomass. Among them, the hot-spot incorporated NDVI ($NDVI_{HS})\;$ proposed by Pocewicz et al. [37] could estimate the above-ground biomass in the most efficient manner (R^2^ = 0.57, RMSE = 55.22). However, it should be noted that none of the extant multi-angular indices were proposed for the estimation of above-ground biomass. The $NDVI_{HS}\;$ was proposed for the estimation of leaf area index. Wang et al. [53] tested angular and band effects on forest biomass retrieval and found that off-nadir vegetation indices could predict the forest biomass more accurately than the nadir. Cui et al. [54] also emphasized typical angle reflectances for estimating canopy heights. In line with these studies, the proposal of VSI for the sole purpose of deriving volumetric structure of the canopy (above-ground biomass) is a timely and significant contribution.

Field survey data are important for biomass modeling research. In this research, above-ground biomass data selected from five forests in the New England region ranged from 18.72 to 309.76 Mg/ha with a mean value of 182.62 Mg/ha, which was considered quite diverse for the assessment of multi-angular vegetation indices. Choi et al. [55] used this dataset to evaluate waveform lidar-based canopy height metrics. Similarly, Park et al. [56] utilized this dataset for examining the canopy heights estimated from waveform lidar data. Wang et al. [57] also utilized this dataset for validating the global estimation of forest canopy height. These research studies show the feasibility of this dataset for assessing the potential of multi-angular vegetation indices.

## 5. Conclusions

In this research, the Vegetation Structure Index (VSI) was proposed based on the concept that higher near-infrared reflectance in the fore-scattering direction indicates exposure of higher contents of the canopy volume, whereas lower red reflectance in the back-scattering direction indicates suppression of the ground reflectance with higher contents of the canopy volume. The VSI was found to be more sensitive to the above-ground biomass in the New England forests than other extant multi-angular vegetation indices. Achieving 7% increase in the estimation of above-ground biomass over the extant multi-angular indices by the VSI is considerable. It suggests that the right choice of the spectral channel and observation geometry should be considered for improving the estimates of the above-ground biomass. In addition, the right choice of seasonal data (summer) was found to be important for estimating the forest biomass while other seasonal data were either insensitive or pointless. The VSI has been derived from the MODIS-based BRDF parameters which can be generated all over the globe. Availability of much higher resolution bi-directional reflectance data is expected in the future for improved estimates of the above-ground biomass in the field of multi-angular satellite remote sensing.

## Figures and Tables

**Table 1:** Overview of the multi-angular vegetation indices available in the literature. The iso, H, N, and D refers to the configuration of the sun-sensor geometries at Nadir-sun Nadir-view, Hot-spot, Nadir, and Dark-spot, respectively.

Multi-Angular Vegetation Indices	Formula	Reference	Target Areas
Nadir BRDF-adjusted NDVI ($NDVI_{iso}$)	$\frac{Nir_{iso}-Red_{iso}}{Nir_{iso}+Red_{iso}}$	Schaaf et al. [33]	Vegetation parameters
Anisotropy index ($ANIX_{Red}$)	$\frac{H_{red}}{D_{red}}$	Sandmeier et al. [34]	Land cover types
Anisotropy index ($ANIX_{Nir}$)	$\frac{H_{nir}}{D_{nir}}$	Sandmeier et al. [34]	Land cover types
Hot-spot dark-spot index ($HDS_{red})$	$\frac{{\;H}_{red}-D_{red}\;}{D_{red}}$	Lacaze et al. [35]	Vegetation clumping
Normalized difference between hot-spot and dark-spot index ($NDHD_{nir}$)	$\frac{{\;H}_{nir}-{\;D}_{nir}}{H_{nir}+D_{nir}}$	Chen et al. [36]	Vegetation clumping
Hot-spot dark-spot NDVI (${NDVI}_{HD}$)	$\frac{{\;H}_{nir}-D_{red}}{H_{nir}+D_{red}}$	Pocewicz et al. [37]	Leaf area index
Hot-spot-incorporated NDVI (${NDVI}_{HS})$	$N_{NDVI}\times (1-H_{red}$)	Pocewicz et al. [37]	Leaf area index

**Table 2:** Performance of existing multi-angular vegetation indices.

Multi-Angular Vegetation Indices	R^2^	RMSE
Anisotropy index ($ANIX_{Red}$)	0.25	73.07
Anisotropy index ($ANIX_{Nir}$)	0.14	78.32
Hot-spot dark spot index ($HDS_{red})$	0.25	73.07
Normalized difference between hot-spot and dark-spot index ($NDHD_{nir}$)	0.17	77.34
Hot-spot dark-spot NDVI (${NDVI}_{HD}$)	0.23	74.24
Hot-spot incorporated NDVI (${NDVI}_{HS})$	0.57	55.52
Nadir BRDF-adjusted NDVI $\left(NDVI_{iso}\right)$	0.54	57.42

**Table 3:** Calculation of nonparametric rank correlation *p*-values.

Multi-Angular Indices and Reflectances	Spearman’s Rank Correlation * p * -Value	Kendall’s Rank Correlation * p * -Value
Anisotropy index ($ANIX_{Red}$)	0.005527	0.008450
Anisotropy index ($ANIX_{Nir}$)	0.015114	0.014109
Hot-spot dark spot index ($HDS_{red})$	0.005527	0.008450
Normalized difference between hot-spot and dark-spot index ($NDHD_{nir}$)	0.009190	0.008829
Hot-spot dark-spot NDVI (${NDVI}_{HD}$)	0.000148	0.001236
Hot-spot incorporated NDVI (${NDVI}_{HS})$	0.000000	0.000000
Nadir BRDF-adjusted NDVI $\left(NDVI_{iso}\right)$	0.000000	0.000000
Near infrared (Back-scattering)	0.000000	0.000001
Near infrared (Nadir)	0.000000	0.000000
Near infrared (Fore-scattering)	0.000000	0.000000
Red (Back-scattering)	0.000001	0.000006
Red (Nadir)	0.000317	0.000333
Red (Fore-scattering)	0.564852	0.692547
Fore-scattering Back-scattering NDVI ($NDVI_{Fore-back}$)	0.000000	0.000000
Vegetation Structure Index (VSI)	0.000000	0.000000

## Data Availability

Not applicable.
